# Supplementary material for: Bulk Operator Reconstruction in Topological Tensor Network and Generalized Free Fields
Source: Entropy (Basel). 2023 Nov 15;25(11):1543. doi: 10.3390/e25111543 (PMC10670195; doi:10.3390/e25111543)
Supplement: Supplementary file 1 [file entropy-25-01543-s001.zip › supplementary-material.pdf]

Supplementary Material for "Bulk Operator Reconstruction in Topological Tensor Network and Generalized Free Fields"

Xiangdong Zeng and Ling-Yan Hung

(Dated: November 10, 2023)

I. SOLUTION FOR 2+1D FIBONACCI MODEL

The general solution for the 2+1d Fibonacci model is given by the linear combination of vectors $v^{(p)}$ in Equation (59) plus the specific solution part $A^{(\mu)}$ for each σ_μ. $A^{(\mu)}$ is an 8×8 matrix whose last 3 columns are zero, i.e.

$$A^{(\mu)} = \begin{pmatrix} \tilde{A}^{(\mu)} & \mathbf{0} & \mathbf{0} & \mathbf{0} \end{pmatrix}, \tag{1}$$

and the 8×5 matrices $\tilde{A}^{(\mu)}$ are given by the following, where $\phi = (1+\sqrt{5})/2$ and $\omega = \mathrm{e}^{\mathrm{i}\pi/5}$:

$$\tilde{A}^{(0)} = \begin{pmatrix} 1 & 0 & 0 & 0 & 0 \\ 0 & 1 & 0 & 0 & 0 \\ 0 & 0 & 1 & 0 & 0 \\ 0 & 0 & 0 & 1 & 0 \\ 0 & 0 & 0 & 0 & 1 \\ 0 & 0 & 1 & 1 & -1 \\ 0 & 1 & 0 & 1 & -1 \\ \phi & \sqrt{\phi} & \sqrt{\phi} & -\frac{1}{\sqrt{\phi}} & \frac{\phi+1}{\sqrt{\phi}} \end{pmatrix}, \qquad \tilde{A}^{(1)} = \begin{pmatrix} 0 & 0 & 0 & 0 & \frac{1}{\phi} \\ 0 & 0 & 1 & 0 & 0 \\ 0 & 0 & 0 & \frac{1}{\sqrt{\phi}} & -\frac{1}{\sqrt{\phi}} \\ \phi^{3/2} & 1 & 0 & 0 & 0 \\ 0 & 1 & 0 & 0 & 0 \\ \phi^{3/2} & 0 & 0 & \frac{1}{\sqrt{\phi}} & -\frac{1}{\sqrt{\phi}} \\ \phi^{3/2} & 0 & 1 & 0 & 0 \\ -\phi & \sqrt{\phi} & \sqrt{\phi} & 1 & 0 \end{pmatrix},$$

$$\tilde{A}^{(2)} = \begin{pmatrix} 0 & \frac{1}{\phi} & 0 & 0 & 0 \\ 0 & 0 & 0 & \frac{1}{\sqrt{\phi}} & -\frac{1}{\sqrt{\phi}} \\ \phi & 0 & 0 & 0 & 0 \\ 0 & 0 & 1 & 0 & \sqrt{\phi} \\ 0 & 0 & 1 & 0 & 0 \\ \phi & 0 & 0 & 0 & \sqrt{\phi} \\ 0 & 0 & 0 & \frac{1}{\sqrt{\phi}} & \frac{\phi-1}{\sqrt{\phi}} \\ \phi^{3/2} & 1 & \sqrt{\phi} & 1 & -2 \end{pmatrix}, \qquad \tilde{A}^{(3)} = \begin{pmatrix} 0 & 0 & \frac{1}{\phi} & 0 & 0 \\ \phi & 0 & 0 & 0 & 0 \\ 0 & 0 & 0 & 0 & 1 \\ 0 & \sqrt{\phi} & 0 & \frac{1}{\sqrt{\phi}} & -\frac{1}{\sqrt{\phi}} \\ 0 & 0 & 0 & \frac{1}{\sqrt{\phi}} & -\frac{1}{\sqrt{\phi}} \\ 0 & \sqrt{\phi} & 0 & 0 & 1 \\ \phi & \sqrt{\phi} & 0 & 0 & 0 \\ \phi^{3/2} & -1 & 1 & 1 & \sqrt{\phi}-1 \end{pmatrix},$$

$$\tilde{A}^{(4)} = \begin{pmatrix} 0 & 0 & 0 & \frac{1}{\phi^{3/2}} & -\frac{1}{\phi^{3/2}} \\ 0 & 0 & 0 & 0 & 1 \\ 0 & 1 & 0 & 0 & 0 \\ \phi & 0 & \sqrt{\phi} & 0 & 0 \\ \phi & 0 & 0 & 0 & 0 \\ 0 & 1 & \sqrt{\phi} & 0 & 0 \\ 0 & 0 & \sqrt{\phi} & 0 & 1 \\ \phi^{3/2} & \sqrt{\phi} & -1 & \frac{1}{\sqrt{\phi}} & \frac{\phi-1}{\sqrt{\phi}} \end{pmatrix}, \qquad \tilde{A}^{(5)} = \begin{pmatrix} 1 & 0 & 0 & 0 & 0 \\ 0 & \omega^4 & 0 & 0 & 0 \\ 0 & 0 & -\omega & 0 & 0 \\ 0 & 0 & 0 & -\omega^3 & \omega^3+\omega^2 \\ 0 & 0 & 0 & 0 & \omega^2 \\ 0 & 0 & -\omega & -\omega^3 & \omega^3 \\ 0 & \omega^4 & 0 & -\omega^3 & \omega^3 \\ \phi & \omega^4\sqrt{\phi} & -\omega\sqrt{\phi} & \frac{\omega^3}{\sqrt{\phi}} & \frac{\omega^2\phi-\omega^3}{\sqrt{\phi}} \end{pmatrix},$$

$$\tilde{A}^{(6)} = \begin{pmatrix} 0 & 0 & 0 & 0 & \frac{\omega^2}{\phi} \\ 0 & 0 & -\omega & 0 & 0 \\ 0 & 0 & 0 & -\frac{\omega^3}{\sqrt{\phi}} & \frac{\omega^3}{\sqrt{\phi}} \\ \phi^{3/2} & \omega^4 & 0 & 0 & 0 \\ 0 & \omega^4 & 0 & 0 & 0 \\ \phi^{3/2} & 0 & 0 & -\frac{\omega^3}{\sqrt{\phi}} & \frac{\omega^3}{\sqrt{\phi}} \\ \phi^{3/2} & 0 & -\omega & 0 & 0 \\ -\phi & \omega^4\sqrt{\phi} & -\omega\sqrt{\phi} & -\omega^3 & \omega^3+\omega^2 \end{pmatrix}, \qquad \tilde{A}^{(7)} = \begin{pmatrix} 0 & \frac{\omega^4}{\phi} & 0 & 0 & 0 \\ 0 & 0 & 0 & -\frac{\omega^3}{\sqrt{\phi}} & \frac{\omega^3}{\sqrt{\phi}} \\ \phi & 0 & 0 & 0 & 0 \\ 0 & 0 & -\omega & 0 & \omega^2\sqrt{\phi} \\ 0 & 0 & -\omega & 0 & 0 \\ \phi & 0 & 0 & 0 & \omega^2\sqrt{\phi} \\ 0 & 0 & 0 & -\frac{\omega^3}{\sqrt{\phi}} & \frac{\omega^3+\omega^2\phi}{\sqrt{\phi}} \\ \phi^{3/2} & \omega^4 & -\omega\sqrt{\phi} & -\omega^3 & \omega^3-\omega^2 \end{pmatrix},$$

$$\tilde{A}^{(8)} = \begin{pmatrix} 0 & 0 & -\frac{\omega}{\phi} & 0 & 0 \\ \phi & 0 & 0 & 0 & 0 \\ 0 & 0 & 0 & 0 & \omega^2 \\ 0 & \omega^4\sqrt{\phi} & 0 & -\frac{\omega^3}{\sqrt{\phi}} & \frac{\omega^3}{\sqrt{\phi}} \\ 0 & 0 & 0 & -\frac{\omega^3}{\sqrt{\phi}} & \frac{\omega^3}{\sqrt{\phi}} \\ 0 & \omega^4\sqrt{\phi} & 0 & 0 & \omega^2 \\ \phi & \omega^4\sqrt{\phi} & 0 & 0 & 0 \\ \phi^{3/2} & -\omega^4 & -\omega & -\omega^3 & \omega^3+\omega^2\sqrt{\phi} \end{pmatrix}, \qquad \tilde{A}^{(9)} = \begin{pmatrix} 0 & 0 & 0 & -\frac{\omega^3}{\phi^{3/2}} & \frac{\omega^3}{\phi^{3/2}} \\ 0 & 0 & 0 & 0 & \omega^2 \\ 0 & \omega^4 & 0 & 0 & 0 \\ \phi & 0 & -\omega\sqrt{\phi} & 0 & 0 \\ \phi & 0 & 0 & 0 & 0 \\ 0 & \omega^4 & -\omega\sqrt{\phi} & 0 & 0 \\ 0 & 0 & -\omega\sqrt{\phi} & 0 & \omega^2 \\ \phi^{3/2} & \omega^4\sqrt{\phi} & \omega & -\frac{\omega^3}{\sqrt{\phi}} & \frac{\omega^3+\omega^2\phi}{\sqrt{\phi}} \end{pmatrix},$$

$$\tilde{A}^{(10)} = \begin{pmatrix} 1 & 0 & 0 & 0 & 0 \\ 0 & -\omega^3 & 0 & 0 & 0 \\ 0 & 0 & \omega^2 & 0 & 0 \\ 0 & 0 & 0 & -\omega & \omega^4+\omega \\ 0 & 0 & 0 & 0 & \omega^4 \\ 0 & 0 & \omega^2 & -\omega & \omega \\ 0 & -\omega^3 & 0 & -\omega & \omega \\ \phi & -\omega^3\sqrt{\phi} & \omega^2\sqrt{\phi} & \frac{\omega}{\sqrt{\phi}} & \frac{\omega^4\phi-\omega}{\sqrt{\phi}} \end{pmatrix}, \qquad \tilde{A}^{(11)} = \begin{pmatrix} 0 & 0 & 0 & 0 & \frac{\omega^4}{\phi} \\ 0 & 0 & \omega^2 & 0 & 0 \\ 0 & 0 & 0 & -\frac{\omega}{\sqrt{\phi}} & \frac{\omega}{\sqrt{\phi}} \\ \phi^{3/2} & -\omega^3 & 0 & 0 & 0 \\ 0 & -\omega^3 & 0 & 0 & 0 \\ \phi^{3/2} & 0 & 0 & -\frac{\omega}{\sqrt{\phi}} & \frac{\omega}{\sqrt{\phi}} \\ \phi^{3/2} & 0 & \omega^2 & 0 & 0 \\ -\phi & -\omega^3\sqrt{\phi} & \omega^2\sqrt{\phi} & -\omega & \omega^4+\omega \end{pmatrix},$$

$$\tilde{A}^{(12)} = \begin{pmatrix} 0 & -\frac{\omega^3}{\phi} & 0 & 0 & 0 \\ 0 & 0 & 0 & -\frac{\omega}{\sqrt{\phi}} & \frac{\omega}{\sqrt{\phi}} \\ \phi & 0 & 0 & 0 & 0 \\ 0 & 0 & \omega^2 & 0 & \omega^4\sqrt{\phi} \\ 0 & 0 & \omega^2 & 0 & 0 \\ \phi & 0 & 0 & 0 & \omega^4\sqrt{\phi} \\ 0 & 0 & 0 & -\frac{\omega}{\sqrt{\phi}} & \frac{\omega+\omega^4\phi}{\sqrt{\phi}} \\ \phi^{3/2} & -\omega^3 & \omega^2\sqrt{\phi} & -\omega & \omega-\omega^4 \end{pmatrix}, \qquad \tilde{A}^{(13)} = \begin{pmatrix} 0 & 0 & \frac{\omega^2}{\phi} & 0 & 0 \\ \phi & 0 & 0 & 0 & 0 \\ 0 & 0 & 0 & 0 & \omega^4 \\ 0 & -\omega^3\sqrt{\phi} & 0 & -\frac{\omega}{\sqrt{\phi}} & \frac{\omega}{\sqrt{\phi}} \\ 0 & 0 & 0 & -\frac{\omega}{\sqrt{\phi}} & \frac{\omega}{\sqrt{\phi}} \\ 0 & -\omega^3\sqrt{\phi} & 0 & 0 & \omega^4 \\ \phi & -\omega^3\sqrt{\phi} & 0 & 0 & 0 \\ \phi^{3/2} & \omega^3 & \omega^2 & -\omega & \omega+\omega^4\sqrt{\phi} \end{pmatrix},$$

$$\tilde{A}^{(14)} = \begin{pmatrix} 0 & 0 & 0 & -\frac{\omega}{\phi^{3/2}} & \frac{\omega}{\phi^{3/2}} \\ 0 & 0 & 0 & 0 & \omega^4 \\ 0 & -\omega^3 & 0 & 0 & 0 \\ \phi & 0 & \omega^2\sqrt{\phi} & 0 & 0 \\ \phi & 0 & 0 & 0 & 0 \\ 0 & -\omega^3 & \omega^2\sqrt{\phi} & 0 & 0 \\ 0 & 0 & \omega^2\sqrt{\phi} & 0 & \omega^4 \\ \phi^{3/2} & -\omega^3\sqrt{\phi} & -\omega^2 & -\frac{\omega}{\sqrt{\phi}} & \frac{\omega+\omega^4\phi}{\sqrt{\phi}} \end{pmatrix}, \qquad \tilde{A}^{(15)} = \begin{pmatrix} 1 & 0 & 0 & 0 & 0 \\ 0 & \omega^2 & 0 & 0 & 0 \\ 0 & 0 & -\omega^3 & 0 & 0 \\ 0 & 0 & 0 & \omega^4 & -\omega^4-\omega \\ 0 & 0 & 0 & 0 & -\omega \\ 0 & 0 & -\omega^3 & \omega^4 & -\omega^4 \\ 0 & \omega^2 & 0 & \omega^4 & -\omega^4 \\ \phi & \omega^2\sqrt{\phi} & -\omega^3\sqrt{\phi} & -\frac{\omega^4}{\sqrt{\phi}} & \frac{\omega^4-\omega\phi}{\sqrt{\phi}} \end{pmatrix},$$

$$\tilde{A}^{(16)} = \begin{pmatrix} 0 & 0 & 0 & 0 & -\frac{\omega}{\phi} \\ 0 & 0 & -\omega^3 & 0 & 0 \\ 0 & 0 & 0 & \frac{\omega^4}{\sqrt{\phi}} & -\frac{\omega^4}{\sqrt{\phi}} \\ \phi^{3/2} & \omega^2 & 0 & 0 & 0 \\ 0 & \omega^2 & 0 & 0 & 0 \\ \phi^{3/2} & 0 & 0 & \frac{\omega^4}{\sqrt{\phi}} & -\frac{\omega^4}{\sqrt{\phi}} \\ \phi^{3/2} & 0 & -\omega^3 & 0 & 0 \\ -\phi & \omega^2\sqrt{\phi} & -\omega^3\sqrt{\phi} & \omega^4 & -\omega^4-\omega \end{pmatrix}, \qquad \tilde{A}^{(17)} = \begin{pmatrix} 0 & \frac{\omega^2}{\phi} & 0 & 0 & 0 \\ 0 & 0 & 0 & \frac{\omega^4}{\sqrt{\phi}} & -\frac{\omega^4}{\sqrt{\phi}} \\ \phi & 0 & 0 & 0 & 0 \\ 0 & 0 & -\omega^3 & 0 & -\omega\sqrt{\phi} \\ 0 & 0 & -\omega^3 & 0 & 0 \\ \phi & 0 & 0 & 0 & -\omega\sqrt{\phi} \\ 0 & 0 & 0 & \frac{\omega^4}{\sqrt{\phi}} & \frac{-\omega^4-\omega\phi}{\sqrt{\phi}} \\ \phi^{3/2} & \omega^2 & -\omega^3\sqrt{\phi} & \omega^4 & \omega-\omega^4 \end{pmatrix},$$

$$\tilde{A}^{(18)} = \begin{pmatrix} 0 & 0 & -\frac{\omega^3}{\phi} & 0 & 0 \\ \phi & 0 & 0 & 0 & 0 \\ 0 & 0 & 0 & 0 & -\omega \\ 0 & \omega^2\sqrt{\phi} & 0 & \frac{\omega^4}{\sqrt{\phi}} & -\frac{\omega^4}{\sqrt{\phi}} \\ 0 & 0 & 0 & \frac{\omega^4}{\sqrt{\phi}} & -\frac{\omega^4}{\sqrt{\phi}} \\ 0 & \omega^2\sqrt{\phi} & 0 & 0 & -\omega \\ \phi & \omega^2\sqrt{\phi} & 0 & 0 & 0 \\ \phi^{3/2} & -\omega^2 & -\omega^3 & \omega^4 & -\omega^4-\omega\sqrt{\phi} \end{pmatrix}, \qquad \tilde{A}^{(19)} = \begin{pmatrix} 0 & 0 & 0 & \frac{\omega^4}{\phi^{3/2}} & -\frac{\omega^4}{\phi^{3/2}} \\ 0 & 0 & 0 & 0 & -\omega \\ 0 & \omega^2 & 0 & 0 & 0 \\ \phi & 0 & -\omega^3\sqrt{\phi} & 0 & 0 \\ \phi & 0 & 0 & 0 & 0 \\ 0 & \omega^2 & -\omega^3\sqrt{\phi} & 0 & 0 \\ 0 & 0 & -\omega^3\sqrt{\phi} & 0 & -\omega \\ \phi^{3/2} & \omega^2\sqrt{\phi} & \omega^3 & \frac{\omega^4}{\sqrt{\phi}} & \frac{-\omega^4-\omega\phi}{\sqrt{\phi}} \end{pmatrix},$$

$$\tilde{A}^{(20)} = \begin{pmatrix} 1 & 0 & 0 & 0 & 0 \\ 0 & -\omega & 0 & 0 & 0 \\ 0 & 0 & \omega^4 & 0 & 0 \\ 0 & 0 & 0 & \omega^2 & -\omega^3-\omega^2 \\ 0 & 0 & 0 & 0 & -\omega^3 \\ 0 & 0 & \omega^4 & \omega^2 & -\omega^2 \\ 0 & -\omega & 0 & \omega^2 & -\omega^2 \\ \phi & -\omega\sqrt{\phi} & \omega^4\sqrt{\phi} & -\frac{\omega^2}{\sqrt{\phi}} & \frac{\omega^2-\omega^3\phi}{\sqrt{\phi}} \end{pmatrix}, \qquad \tilde{A}^{(21)} = \begin{pmatrix} 0 & 0 & 0 & 0 & -\frac{\omega^3}{\phi} \\ 0 & 0 & \omega^4 & 0 & 0 \\ 0 & 0 & 0 & \frac{\omega^2}{\sqrt{\phi}} & -\frac{\omega^2}{\sqrt{\phi}} \\ \phi^{3/2} & -\omega & 0 & 0 & 0 \\ 0 & -\omega & 0 & 0 & 0 \\ \phi^{3/2} & 0 & 0 & \frac{\omega^2}{\sqrt{\phi}} & -\frac{\omega^2}{\sqrt{\phi}} \\ \phi^{3/2} & 0 & \omega^4 & 0 & 0 \\ -\phi & -\omega\sqrt{\phi} & \omega^4\sqrt{\phi} & \omega^2 & -\omega^3-\omega^2 \end{pmatrix},$$

$$\tilde{A}^{(22)} = \begin{pmatrix} 0 & -\frac{\omega}{\phi} & 0 & 0 & 0 \\ 0 & 0 & 0 & \frac{\omega^2}{\sqrt{\phi}} & -\frac{\omega^2}{\sqrt{\phi}} \\ \phi & 0 & 0 & 0 & 0 \\ 0 & 0 & \omega^4 & 0 & -\omega^3\sqrt{\phi} \\ 0 & 0 & \omega^4 & 0 & 0 \\ \phi & 0 & 0 & 0 & -\omega^3\sqrt{\phi} \\ 0 & 0 & 0 & \frac{\omega^2}{\sqrt{\phi}} & \frac{-\omega^3\phi-\omega^2}{\sqrt{\phi}} \\ \phi^{3/2} & -\omega & \omega^4\sqrt{\phi} & \omega^2 & \omega^3-\omega^2 \end{pmatrix}, \qquad \tilde{A}^{(23)} = \begin{pmatrix} 0 & 0 & \frac{\omega^4}{\phi} & 0 & 0 \\ \phi & 0 & 0 & 0 & 0 \\ 0 & 0 & 0 & 0 & -\omega^3 \\ 0 & -\omega\sqrt{\phi} & 0 & \frac{\omega^2}{\sqrt{\phi}} & -\frac{\omega^2}{\sqrt{\phi}} \\ 0 & 0 & 0 & \frac{\omega^2}{\sqrt{\phi}} & -\frac{\omega^2}{\sqrt{\phi}} \\ 0 & -\omega\sqrt{\phi} & 0 & 0 & -\omega^3 \\ \phi & -\omega\sqrt{\phi} & 0 & 0 & 0 \\ \phi^{3/2} & \omega & \omega^4 & \omega^2 & -\omega^3\sqrt{\phi}-\omega^2 \end{pmatrix},$$

$$\tilde{A}^{(24)} = \begin{pmatrix} 0 & 0 & 0 & \frac{\omega^2}{\phi^{3/2}} & -\frac{\omega^2}{\phi^{3/2}} \\ 0 & 0 & 0 & 0 & -\omega^3 \\ 0 & -\omega & 0 & 0 & 0 \\ \phi & 0 & \omega^4\sqrt{\phi} & 0 & 0 \\ \phi & 0 & 0 & 0 & 0 \\ 0 & -\omega & \omega^4\sqrt{\phi} & 0 & 0 \\ 0 & 0 & \omega^4\sqrt{\phi} & 0 & -\omega^3 \\ \phi^{3/2} & -\omega\sqrt{\phi} & -\omega^4 & \frac{\omega^2}{\sqrt{\phi}} & \frac{-\omega^3\phi-\omega^2}{\sqrt{\phi}} \end{pmatrix}. \tag{2}$$
